# Some Observations upon Pyorrhœa Alveolaris

**Published:** 1894-04

**Authors:** R. Ottolengui

**Affiliations:** New York


### SOME OBSERVATIONS UPON PYORRHŒA ALVEOLARIS. REVIEW OF PROFESSOR PEIRCE'S PAPER.[1]

BY R. OTTOLENGUI, M.D.S., NEW YORK.

I HAVE been requested to send you my opinions upon pyorrhœa alveolaris, viewed from the stand-point of an article by Professor C. N. Peirce, in the INTERNATIONAL DENTAL JOURNAL for January. In the same number of the JOURNAL I find an article upon the same subject by Dr. F. T. Van Woert, together with the discussion of his paper, and in the February number I find the discussion of Dr. Peirce's article. With your permission, therefore, I will take up the subject from the stand-point of the papers and discussion found in both numbers of the JOURNAL.

---

[1] Read before the Eighth District Dental Society, Buffalo, N. Y., February 26, 1894.

It is no new idea that pyorrhœa is attributable to a systemic rather than to a local cause. More than ten years ago, Dr. William H. Atkinson, when treating these cases, prescribed constitutional remedies, cinchonidia being one of his dependencies. The new thought in the paper by Professor Peirce is that uric acid is a primary cause in the production of the worst form of this disease. To Dr. Peirce we must give full credit for his published work, and for the series of investigations and experiments that have led up to it. If I mention, therefore, that this idea is not a new one to me, it is not to lessen the force of what Professor Peirce has given us, but rather to show that what opinions I may express may have more value than if it were true that it has only been since a few weeks ago that I have given this matter thought.

I think it is fully five years ago since Dr. M. L. Rhein first told me that, in his opinion, there was a close relationship between the most aggravated (and, I may say, aggravating) form of this disease and the gouty diathesis. From that time I have been seeking closely for histories which connect this disease with the gouty complaints. I also know that during this time it has been a frequent habit of Dr. Rhein to have examinations of urine made where the disease was conspicuously present. I regret to say that I cannot give any results that he has deduced, nor would it be proper for me to anticipate what he has the right to publish as his own. But I would suggest that, as time enough will elapse after receiving this and before your meeting, Dr. Rhein be invited to give you his views upon this subject.

I will only mention, in relation to what I have observed myself, that, whilst I have no clinical facts to offer, it is true that, though I accepted Dr. Rhein's view as probably correct, I have been unable to verify the fact. I have questioned over three hundred persons afflicted with virulent pyorrhœal conditions, and the number who have admitted that they had suffered from any form of disease attributable to gouty diathesis was not at all commensurate with the number we would expect to find were this the true and only explanation. However, it is quite possible that even in those cases in which there was no known history of rheumatic tendency uric acid may have been the most important factor in causing the local disease symptoms about the teeth. What more I could say would be conjectural, and therefore unprofitable. I am inclined to view the theory as probable, but it is scarcely time for its free adoption. There is too great a tendency among dentists to adopt every new idea, and the result is that many so hastily accepted must eventually be discarded. I think that Professor Peirce is working in the right direction. He has the ability to pursue the subject to a satisfactory and conclusive end, but I believe that it will be wise to wait a while before announcing to our patients that at last we know all about it.

I would next like to say a word about terminology. Pyorrhœa alveolaris is a bad name. It is indefinite, and almost meaningless for the purpose to which it has been applied. But there is this in favor of it: it is already well known to a vast number of laymen. It is not at all uncommon for me to have new patients ask me to examine their mouths to see if they have pyorrhœa. They usually use the one word, which is significant. The public wants simple names for their diseases. Since there is no other part of the body which is afflicted with pyorrhœa, it is clear that the explanatory word alveolaris is unnecessary. Therefore I would favor the adoption of the name pyorrhœa as a designation for the class. Next, we recognize that there is more than one form of the disease, and I am not sure that there may not be more than two distinct forms, with different etiology. I do not favor the names proposed by Dr. Black, nor do I like those suggested by Professor Peirce, but I cannot offer any better. Perhaps it might do to call the worst form serumal pyorrhœa and the other calcic. But I do not really advocate this, because it is still to be decided what part the deposits play in the disease, there being no doubt in my mind that it may exist in its most discouraging form without deposits either from the blood or from the saliva.

From the realm of my experience, I will endeavor to describe some distinct forms of the disease, as I have seen them, together with concomitant peculiarities.

We frequently hear of men who effect radical cures of pyorrhœa. I doubt very much that these men have done so. I have listened over and over to their histories of such cases, and I have read reports that have been printed, but almost invariably I have been impressed by the thought that these men have been incompetent diagosticians. To them, any ooze of pus from the pocket around the neck of a tooth is a true case. Why not? Is it not pyorrhœa alveolaris?—pus from the alveolus? But however poor they may be as diagnosticians, we cannot deny the honesty of their reports. Therefore we must recognize that there is a pus-generating disease, which is curable.

I should not include this in the class which I would denominate pyorrhœa, or if I did I think I should call it gingival pyorrhœa, for, first, it is a disease confined strictly to the gingivæ, and, secondly, it is not caused by calcic deposits, though the deposits play an important part, as I shall describe.

Gingival pyorrhœa (to use the term as a temporary designation, for the purposes of this paper) is really a gingivitis which has been neglected until a suppurative stage is reached. This suppuration may result in two ways, or may be caused by both together. The first appearance is a localized, but well-marked inflammation, confined to the gingival pedicle which lies between the teeth. For a long period, it will extend no farther, and there will present a well-defined line, which appears to separate the inflamed from the normal tissue. This line will be just below one drawn across the highest point of the gingival arches. If the patient notices the disturbance at all, the report will be "My teeth bleed when I clean them." In this stage the disease is not difficult to control. Nightly applications of glycerole of tannin will accomplish much. In time the inflamed territory expands, and the disease spreads along the arch of the gingiva, until the whole of the margin of the gum is involved. Then the gums appear turgid and swelled. They are also rough, and loosely bind the teeth. A sort of pocket is formed and deposits are invited. These in turn add to the trouble, until we find them exerting an irritating influence, which ends in a purulent condition. Or, where deposits are not made, the gradual degeneration of the vitality of the part will result in pus. In these cases, pus may be abundant, but the pocket will not be deep, and the pericementum is not at all involved. Of this I am positive. When the pericementum is involved, then I would say that from gingival pyorrhœa we have supervening the condition which I have denominated salivary pyorrhœa. I do not mean that this second form always originates in a gingivitis, but where a gingivitis is present we have a fruitful soil for the grafting of the more dangerous malady.

The salivary pyorrhœa, then, is where salivary calculus is found about the necks of the teeth, with pockets extending up and encroaching upon the area originally occupied by the alveolar bone, and from these pockets we expect pus. This condition I would include among those possibly attributable to uric acid, though the next is more probably so.

Where the disease is solely serumal pyorrhœa, we would argue that salivary calculus was not necessarily present at the initiatory period. The deposit has been made at or near the foraminal end of the tooth, and the subsequent pocket has been formed by the burrowing of the pus which has accumulated as a result of irritation. I am inclined to believe that in these cases the serumal deposit is not a mere incident of the disease, but that it is the primary lesion. If not, then we must imagine that the disease exists first; that we have pus accumulating at the end of the tooth and burrowing its way outward until a pocket has been formed, and that the deposits occur afterwards. Now, who can truthfully report such a condition? We have seen pus discharges where no deposits exist, but who can assert that deposits of one kind or the other have not existed prior to the examination made by himself? Personally I have never noted such a condition, except where, instead of a well-defined pocket, there has been rather a well-marked separation of the soft tissues from the tooth itself, with a dissolution of the bone; and invariably I have observed that the pockets do not reach the end of the root, except in the final stages, when extraction becomes almost an immediate necessity.

The serumal pyorrhœa, then, in my opinion, begins when the deposit is made at the end of the root.

But next we have complex conditions. The serumal pyorrhœa may become engrafted upon the salivary. That is, the saliva may deposit calculi first, and in an advanced stage of that disease we may have serumal deposits occurring. Contrarily, the serumal deposits may present first, and after a pocket has been made it is easy to imagine the salivary deposits occurring. Whichever way the final condition may come about, the result is the same,—a combination of both diseases. That uric acid is an operating cause here is more probable than with any of the other conditions.

By what name shall we designate that other dreadful form, where no deposits are present? Once more maintaining my point that the pocket begins at the neck of the tooth and increases towards the end of the root, I will ask you to consider the following explanation of the disease: Any condition of health which will deplete the system and cause anæmia will superinduce a condition about the teeth favorable to the advancement of this form of pyorrhœa. As a local degenerator, I would point to excessive smoking. The constant high temperature may be said to cook the soft tissues to a point where their vital resistance is materially lessened. There is a waste of tissue in all parts of the body because of use. This waste is restored by vital processes of reorganization, and where the vitality is sapped we can easily imagine a general depletion ensuing, with lack of reparative energy. The soft tissues, and also the bone about the teeth, slowly waste. The gums shrink.

They do not recede at first, but they shrivel, their bony support having also melted away. Pus follows. In this condition recession begins. This recession is more marked about the superior molars, and on the palatal side. This is due to the lack of resistance offered to the action of the tongue pushing the food between the teeth in mastication. The force thus exerted wears away the soft tissues at these points. We may or we may not have pus, and its presence is possibly due to bacterial infection. It is well known that bacteria may be present and do no harm until such time when the vital resistance is lessened, or the temperature increased to a point conducive to their propagation. The first condition we would get in either sex from any constitutional ailment resulting in anæmia, and we might have both were the smoking habit excessively indulged in. What part the nicotine would play as a germicide and how it might act as a virulent poison are points worthy of study. These cases, as unpromising as they appear, are exactly the ones which often result in what seem to be marvellous cures. If the general health of the individual is restored, either by medical aid or by change of scene and habit, the local disturbance not infrequently will disappear, and though the gums and much of the bone have been lost, the teeth become firm again.

I have already written much more than I at first intended, and I must therefore allude rather briefly to the points in Dr. Van Woert's paper which attract my attention. He tells us that necrosis is not an accompanying symptom of pyorrhœa, while it has been long claimed that it is. It seems to me that the advance made by the doctor's investigation is more imaginary than real. I do not doubt that technically he is right, and that true necrosis is not commonly to be found in this disease. But there is no doubt that there is a destruction of the alveolar bone, and whether we call it necrosis, caries, dissolution, or absorption is of small consequence. I would take issue with the doctor, however, where he likens the process of destruction to what he calls the absorption of bone which follows the extraction of the teeth. He has made sections of the bone to prove his point. But I too have made sections of bone, and where but one tooth has been extracted, instead of any absorption of the bone, I have found rather an obliteration of the tooth-socket by a deposition of new bone, which is more cancellous than the normal process. Absorption of the alveolar ridge is commonly found only where several teeth have been taken from one locality, and is subsequent to the healing process, or, if coincident with it, it is due to the fact that the destruction of bone has been great enough to allow a collapse of the remaining walls, so that, when healed, the ridge is shortened. But in pyorrhœa, we have a distinct destruction of the bone, which, if asked to denominate, I should call caries, due to the presence of pus and pressure therefrom.

This brings me to a proposition which I have not seen considered, and which I offer for your future observation and study.

My question is, Whence the pus which accompanies this disease? Is it from the pericementum, or is it from a true pus-generating tissue which replaces the lost alveolus?

In many cases, I have been tempted to believe that the latter is the case; that the pus exudes from the soft tissues overlying the root, and not from the pericementum. If this be true, then we cannot use the term pericementitis. I do not mean that pus never comes from the pericementum, but that in the worst cases it comes chiefly from an opposite direction.

I believe that I have already said enough, as probably you have others to contribute to the discussion, though I had originally intended to touch upon the subject of medicaments. I will, however, postpone that for another occasion.

